# Methodological Issues in Taquet et al.‘s analysis preclude any conclusions regarding AS01 adjuvant’s specific role in dementia prevention

**DOI:** 10.1038/s41541-025-01309-4

**Published:** 2025-12-05

**Authors:** S. Elizabeth Williams, Kate Luisi, Caihua Liang, Alejandro Cane, Elizabeth Begier

**Affiliations:** 1 Pfizer, Inc, New York, New York, NY USA; 2 Pfizer, Inc, Dublin, Ireland

**Keywords:** Diseases, Health care, Immunology, Medical research, Neurology, Neuroscience

## Abstract

Taquet et al. evaluated the impact of AS01-adjuvanted vaccines on subsequent dementia diagnosis^1^. The authors conclude: “No difference was observed between the two AS01-adjuvanted vaccines, suggesting that the AS01 adjuvant itself plays a direct role in lowering dementia risk”. Although the study offers promising evidence, the inference regarding the role of AS01 adjuvant in dementia prevention is not convincingly supported by the presented data or other published literature^2^.

The study employed a retrospective cohort study design using US electronic health record (EHR) data to compare the risk of new dementia diagnosis within 18 months following vaccination with the AS01-adjuvanted herpes zoster vaccine (against shingles) and the “AS01-adjuvanted” RSV vaccine to that with the influenza vaccine (which is not AS01-adjuvanted) (Table 1). AS01-adjuvanted vaccine recipients were matched to an equal number of people who received the influenza vaccine. The study had several strengths, including large sample sizes and propensity score matching to reduce confounding. However, several limitations restrict the ability to draw conclusions about the specific role of the AS01 adjuvant in dementia prevention.

**Table 1:** Selected vaccine-associated risk reduction estimates for new dementia diagnosis among adults

Author (year)	Vaccine	Comparison	Effect Size	95% CI
Herpes Zoster				
Taquet^1^	AS01-adjuvanted HZ	Influenza	0.82 (RMTL ratio)	0.74–0.91
Taquet^8^	AS01-adjuvanted HZ recombinant	Influenza	0.77 (RMTL ratio)	0.75–0.79
Taquet^8^	HZ live vaccine	Influenza	0.86 (RMTL ratio)	0.81–0.91
Wu^2^	HZ [AS01-adjuvanted HZ recombinant and live attenuated virus combined]	Non-vaccinated population	0.69 (HR)	0.66–0.73
Wiemken^14^	HZ [AS01-adjuvanted HZ recombinant and live attenuated virus combined]	Non-vaccinated population	0.75 (HR)	0.71–0.79
Vaccines for other pathogens				
Wu^2^	Any [Mixed types]	Non-vaccinated population	0.65 (HR) Pooled results	0.60–0.71
Taquet^1^	“AS01-adjuvanted” RSV	Influenza	0.71 (RMTL ratio)	0.61–0.83
Wu^2^	Influenza^a^	Non-vaccinated population	0.74 (HR)	0.63–0.87
Wu^2^	TdaP	Non-vaccinated population	0.69 (HR)	0.58–0.82
Wiemken^14^	TdaP	Non-vaccinated population	0.82 (HR)	0.76–0.89

*HZ* herpes zoster, *RMTL* restricted mean time lost, *HR* hazard ratio.

^a^Heterogeneity may exist with various types of adjuvants used in influenza vaccine formulations.

First, only 44% of RSV vaccine exposures had the brand specified, and the authors estimated that 24% of the “AS01-adjuvanted” RSV vaccine group actually received the bivalent RSVpreF vaccine (*Abrysvo*) rather than the adjuvanted RSVpreF3 (*Arexvy*). These vaccines are both protein subunit vaccines based on RSVpreF antigen. RSVpreF3 is based on the original RSVpreF antigen structure derived from an RSV A strain by the US National Institute of Health^3,4^ and contains the AS01 adjuvant. RSVpreF contains the contemporaneous RSVpreF antigens from both RSV A and B strains and is not adjuvanted^5^. Taquet et al. argued that including RSVpreF in their analysis would bias the estimates towards the null, leading to an underestimation of dementia risk reduction. However, if the RSVpreF antigen was the component associated with dementia risk reduction rather than ASO1, its inclusion would not impact the observed protective effect of AS01-adjuvanted RSVpreF3 vaccination.

Second, Taquet et al. did not report any stratified analysis by RSV vaccine type. Such a comparison would have more directly tested the hypotheses that the AS01 adjuvant specifically reduced dementia risk and that grouping RSVPreF and RSVpreF3 together would bias the effect estimates toward the null. Further, while the AS01 dose in the RSVpreF3^6^ is half that of AS01-adjuvanted herpes zoster vaccine^7^, the dementia risk reduction estimate was similar for the two vaccines, indicating no evidence for a dose-response relationship with this vaccine component.

Third, other published literature does not support vaccine-associated dementia risk reduction as being specifically related to the AS01 adjuvant or other adjuvants. A recent systematic review and meta-analysis investigated the association between adult vaccination and dementia risk^2^. The review included 17 studies with almost 2 million participants. Pooled results found that overall, vaccinations were associated with a 35% lower dementia risk (HR = 0.65, 95% CI: 0.60–0.71) and the trend was consistent regardless of vaccination type, with several being significantly associated with decreased risk (Table 1). The effect size is similar to that previously reported by Taquet et al.^8^, where both live and recombinant herpes zoster vaccines were linked with lower dementia risk. The publication also reported a potential dose response relationship where individuals with more complete vaccination status (i.e., having received more than one recommended adult vaccination), and individuals with a history of more annual influenza vaccinations were less likely to develop dementia. Although this review did not specifically compare adjuvanted versus unadjuvanted vaccines, the data suggest that a variety of vaccines had significantly protective effects regardless of vaccine type or inclusion of adjuvant.

Given similar risk reductions seen across multiple vaccine types, it is possible that the observed dementia risk reduction is due to vaccinations’ prevention of severe disease and associated hospitalizations generally. A previously undiagnosed patient with mild cognitive impairment may acutely worsen cognitively after experiencing vaccine-preventable severe disease. Such decompositions are common during acute illnesses in the natural history of dementia^9,10^. Further, a prior history of RSV infection has been specifically linked to increased risk of cognitive impairment. A 2024 population-based study found RSV seropositivity was associated with a twofold increase in mild cognitive impairment and dementia^11^.

Lastly, residual confounding could influence Taquet et al.’s results as well as the other published results summarized above because of these studies’ observational design. To our knowledge, no randomized study has evaluated the association between vaccination and dementia risk and thus, residual confounding (e.g, socioeconomic factors limiting access to vaccination and health-seeking behaviors that increase vaccine uptake^12,13^) cannot be ruled out as contributing to these findings.

## Conclusion

Although this study supports the importance of vaccination in the prevention of dementia, the inference that one component of a vaccine formulation is leading to a reduction in risk of dementia is premature. We agree that this work should be considered hypothesis-generating, requiring additional evaluation. Randomized assessments of the impact of single vaccine components are needed before such conclusions can be drawn.

## Data Availability

No datasets were generated or analyzed during the current study.
